# Reciprocal Influence of Protein Domains in the Cold-Adapted Acyl Aminoacyl Peptidase from *Sporosarcina psychrophila*

**DOI:** 10.1371/journal.pone.0056254

**Published:** 2013-02-15

**Authors:** Federica Parravicini, Antonino Natalello, Elena Papaleo, Luca De Gioia, Silvia Maria Doglia, Marina Lotti, Stefania Brocca

**Affiliations:** Department of Biotechnology and Biosciences, University of Milano-Bicocca, Milan, Italy; University of Queensland, Australia

## Abstract

Acyl aminoacyl peptidases are two-domain proteins composed by a C-terminal catalytic α/β-hydrolase domain and by an N-terminal β-propeller domain connected through a structural element that is at the N-terminus in sequence but participates in the 3D structure of the C-domain. We investigated about the structural and functional interplay between the two domains and the bridge structure (in this case a single helix named α1-helix) in the cold-adapted enzyme from *Sporosarcina psychrophila* (SpAAP) using both protein variants in which entire domains were deleted and proteins carrying substitutions in the α1-helix. We found that in this enzyme the inter-domain connection dramatically affects the stability of both the whole enzyme and the β-propeller. The α1-helix is required for the stability of the intact protein, as in other enzymes of the same family; however in this psychrophilic enzyme only, it destabilizes the isolated β-propeller. A single charged residue (E10) in the α1-helix plays a major role for the stability of the whole structure. Overall, a strict interaction of the SpAAP domains seems to be mandatory for the preservation of their reciprocal structural integrity and may witness their co-evolution.

## Introduction

Acyl aminoacyl peptidases (AAP) are members of the prolyl oligopeptidase (POP) family of serine peptidases and catalyze the removal of N-acylated amino acids from blocked peptides [1]. These enzymes, also referred to as “oxidized protein hydrolases”, are ubiquitous [2], [3], [4] and play a key role in the clearance of cytotoxic denatured proteins [5], [6], [7], [8]. Like all members of the POP family, AAPs contain a catalytic site identical to that of serine peptidases, but they hydrolyze short peptides only. They also cleave acyl chains from esters and are therefore considered *promiscuous* [9]. From a structural point of view, members of the POP family consist of a C-terminal catalytic domain and of an N-terminal β-propeller domain that hides the active site. The two domains are structurally bridged through the N-terminus of the sequence that, however, participates in the structure of the C-terminal catalytic domain. In proteins of the AAP subfamily this region forms a single α-helix (α1) [10], [11].

The architecture of the catalytic domain conforms to the canonical α/β-hydrolases fold common to different hydrolytic enzymes [12]. As for this moiety, AAPs are very close to esterases and lipases, sharing the same sequence order of catalytic residues (Ser - Asp - His) and the Gly-X-Ser-X-Gly motif conserved around the catalytic serine [13]. The β-propeller domain occurs in different protein structures, for example in the cytochrome cd1, the β subunit of the G protein, neuraminidase and sialidase [14]. This fold is highly symmetrical and it is based on repeats [4], [5], [6], [7], [8] of a four-stranded antiparallel β-sheet motif, radially arranged around a central tunnel. The stability of the propeller structure depends on the architecture of its “closure”, that is the way the N- and C-termini are joined. The N- and C-termini can be interdigitated in the same terminal blade of larger domains (the so called “molecular velcro”), or linked by disulphide bonds as it occurs in smaller, four-bladed domains. In the case of prolyl oligopeptidases, however, the circular structure is not “velcroed” nor it is stabilized by disulphide bonds [15] and the two ends of the propeller belong to the catalytic domain [14], [15]. Such a non-velcroed topology was predicted to be structurally weak and flexible enough to allow the central cavity to open. On this basis it was hypothesized that the propeller can act as a gating filter selective towards peptide substrates [16]. Experimental studies demonstrated instead that isolated, unclosed β-propellers can be stable and suggested that enzyme activity relies rather on concerted movements of the peptidase and propeller domains [16], [17]. Nowadays a clamshell-like movement has been proposed to describe the substrate-induced conformational changes performed by several prolyl peptidases [18], [19], although analysis of molecular dynamics reveals that such conformational changes can be strikingly different in different enzymes, leading to different selectivity towards the substrate [20].

Among the small set of AAPs characterized to date, only a few are from Archaea [2], [21], [22] and only one from Eubacteria [23]. The only 3D structure available is that of the hyperthermophilic enzyme from the archaebacterium *Aeropyrum pernix* K1 (ApAAP), a homodimer with each subunit made up of a seven-bladed β-propeller domain and a peptidase domain [21]. In this enzyme, deletion of the N-terminal extension affects the hyperthermostability, the conformational flexibility of the protein and the temperature-dependence of activity [10], but not the overall three-dimensional structure [24].

We have previously described a psychrophilic AAP isolated from the gram-positive *Sporosarcina psychrophila* [23]. This protein (SpAAP) exhibits the typical features of cold-adapted enzymes, since it retains activity at low temperature (10–15% activity at 6°C) and has poor thermal stability. SpAAP hydrolyzes both soluble fatty acid esters and N-acylated amino acid derivatives, with preference for short-chain esters and leucine derivatives, respectively, and displays the same temperature dependence and stability with both substrates [23]. To study the interplay between protein domains in the context of a cold-adapted AAP, we produced the N- and the C-domain in isolation and the whole SpAAP as well as the β-propeller deprived of the first 14 amino-acid residues (including the first α-helix) and proteins carrying single substitutions in α1. We report that the presence of the β-propeller is essential for the structural and functional integrity of the catalytic moiety and that the α1-helix is of paramount relevance for the stability of this protein. Moreover, the α1-helix destabilizes the isolated β-propeller domain, a feature up to date observed only in this protein. The results of this study suggest that in the cold-adapted AAP the interplay of its two domains may fulfil inescapable requirements for the preservation of their structural integrity and may witness their co-evolution. We hypothesize that this strong *liaison* between the structural integrity of catalytic and regulatory domains might be the molecular mechanism selected during evolution to guarantee the functional association between them.

## Materials and Methods

### Modelling and Molecular Dynamics (MD) Simulations

The multiple sequence alignment for protein structure prediction was obtained by the HHPred Server [25] and compared with the results of threading methods, as Phyre [26] and GeneSilico Metaserver [27]. The optimization of the multiple alignments for 3D modeling was carried out by hand, according to information on functional and conserved residues, and secondary structures. The SpAAP model was generated with *MODELLER* version 9.8 [28] using as a template the ApAAP structure (PDB entry 1VE6) [21], which shares with SpAAP 20% and 54% overall sequence identity and similarity, respectively. The model quality was evaluated using Procheck [29], Verify-3D [30] and VADAR [31]. The 3D model was further refined by molecular dynamics (MD) simulations using *GROMACS* 4 and Gromos96 force field (www.gromacs.org). Productive MD simulations were carried out in the isothermal-isobaric ensemble (NPT, 290 K, 1 bar and 2 fs time-step) for 60 ns in explicit solvent with a dodecahedral box characterized by a minimum distance between the solute and the box of 0.6 nm and employing periodic boundary conditions. The systems were equilibrated in several steps of solvent equilibration, thermalization and pressurization (100 ps MD run for each step) before starting the productive runs. Electrostatic interactions were calculated using the Particle-mesh Ewald summation scheme. Van der Waals and Coulomb interactions were truncated at 1.0 nm and conformations stored every 4 ps. The secondary structure content was calculated by the DSSP program [32]. A distance cut-off of 0.6 nm between the charged groups was applied to analyze salt-bridge interactions.

### Strains, Growth Media and Materials

*Escherichia coli* strain DH5α™ (Invitrogen) was used as the host for DNA amplification, whereas strain BL21 (DE3) (EMD Millipore) was the host for heterologous expression. *E. coli* cells were grown in low-salt Luria-Bertani (ls-LB) medium (10 g peptone, 5 g yeast extract, 5 g NaCl in 1 L water) and transformants were selected on agarized plates of ls-LB supplemented with 100 mg/L ampicillin. Oligonucleotides and substrates for esterase activity assays, *p*-nitrophenyl butyrate (*p*NP-But) and *p*-nitrophenyl caprilate (*p*NP-Cap), were from Sigma. Substrates for peptidase activity assays, N-acetyl-L-leucine-*p*-nitroanilide (Nac-leu-*p*NA) and N-acetyl-L-phenylalanine-*p*-nitroanilide (Nac-phe-*p*NA), were from Bachem.

### Cloning and Mutagenesis

Standard recombinant DNA techniques were applied according to Sambrook et al. [33]. Deletions of the SpAAP gene were obtained by back-to-back PCR of plasmid pET22[SpAAP] [23] or, as in the case of the triple mutant K6A_E10A_R14A, by amplification of the vector pET22[SpAAP^K6A_E10A^]. Blunt-end amplimers obtained by back-to-back PCR with non-overlapping phosphorylated oligonucleotides [34] were directly ligated resulting in circularisation of plasmid DNA. Some of the primers were designed to contain, besides the target mutation, a silent mutation introducing or deleting a diagnostic restriction site (shaded in Table S1 - Supporting Information). Phosphorylation of oligonucleotides was carried out by incubation with polynucleotide kinase A (New England Biolabs) at 37°C for 1 h. PCR was carried out with 10 ng of plasmid DNA as a template and each primer at a concentration of 0.5 µM. Sequences of the oligonucleotides employed in this work are reported in Table S1.

PCR was performed in a volume of 50 µl using 1 µl of the high-fidelity *Pfu*II Ultra DNA polymerase (Stratagene) according to the manufacturer’s instructions and applying the following temperature program: 2 min denaturation at 95°C, 30 cycles of 20 s at 95°C/30 s at 50–65°C/4 min at 72°C, and a final extension step of 10 min at 72°C. Following amplification, the template DNA was digested with *Dpn*I and the amplified DNA, purified by ethanol precipitation, was circularized by self ligation with T4 DNA ligase (New England Biolabs) and used to transform DH5α *E. coli* cells. Plasmid constructs were checked by restriction enzymes (New England Biolabs) and DNA sequencing (Primm).

### Expression and Purification of SpAAP Variants

Production of recombinant proteins was carried out growing transformed cells over night in autoinduction ZYM-5052 medium [35] at 25°C by shaking at 220 rpm. In analytical experiments intended to assess the expression of SpAAp variants, after cell harvesting by centrifugation at 1600 *g* at 4°C, samples were frozen at −20°C and the procedure of protein extraction and separation of soluble and insoluble fractions was carried out at room temperature. In detail, thawed pellets were re-suspended in lysis buffer (50 mM sodium phosphate pH 8.0, 300 mM NaCl) added of protease inhibitor cocktail (Sigma). Cell density of different samples was normalized to 8 OD_600_/ml by using variable volumes of buffer. Aliquots of 500 µl were sonicated on ice and centrifuged at 15,550 *g* for 10 min to separate insoluble proteins from the soluble protein fraction. Soluble proteins samples were prepared by mixing the supernatant with the appropriate volume of 4× SDS loading buffer. A volume of 500 µl of 1x SDS loading buffer was used to re-suspend the insoluble pellets. For the preparation of total cell extracts, 100 µl of intact cells re-suspended in lysis buffer were centrifuged at 15,550 *g* for 10 min and the pellet lysed in 100 µl of 1× SDS loading buffer. Sample of soluble, insoluble and total proteins corresponding to the same amount of cells (0.12 OD_600_) were loaded on 15-well minigels.

Protein preparations for biochemical and biophysical assays were obtained from cell pellets immediately after harvesting and the procedures of protein extraction, purification and desalting were carried out at 4°C. Typically, proteins were purified from cells harvested from 50-ml culture, re-suspended in Purification Buffer (PB - 50 mM sodium phosphate pH 8.0, 300 mM NaCl and 10 mM imidazole) and lysed using a cell disruptor (Constant Systems Ltd) at 25,000 psi. Insoluble proteins were separated from the soluble fraction by centrifugation for 10 min at 15550 *g*, at 4°C. The recombinant, his-tagged proteins were purified from the supernatant by immobilized-metal affinity chromatography on Ni^2+^/NTA beads. The clear lysate was loaded on a column containing 1 ml of HIS-Select Nickel Affinity Gel (Sigma) equilibrated with 2 ml of PB containing 10 mM imidazole. The column was washed with 3 ml of PB containing 20 mM imidazole and proteins eluted with PB containing 250 mM imidazole. Protein-containing fractions were exchanged to the final buffer (10 mM sodium phosphate, pH 7.5 or 50 mM sodium phosphate, pH 7.5) by two consecutive gel filtrations on *PD-10* columns (GE Healthcare) according to the manufacturer’s instructions. Protein concentration was determined by the Bradford protein assay (Bio-Rad), using bovine serum albumin as a standard.

SDS-PAGE analyses were carried out on 12% acrylamide Laemmli gels [36] stained with *GelCode Blue* (Pierce) after electrophoresis. Broad-range, pre-stained molecular-weight markers (GeneSpin) were used as standards.

### Activity Assays

Spectrophotometric assays were carried out on *p*NP-But and *p*NP-Cap to measure esterase activity and on Nac-leu-*p*NA and Nac-phe-*p*NA for peptidase activity as previously described [23], with the only exception that reactions were carried out in 10 or 50 mM sodium phosphate buffer, pH 8.5. The effect of temperature on activity was evaluated by measuring at room temperature the residual activity of the enzyme incubated at 50°C for different time periods. All measurements were made in triplicate.

### Circular Dichroism Spectroscopy

CD spectra were recorded on a spectropolarimeter J-815 (JASCO) in a 1-mm pathlength cuvette at room temperature. Samples were in 10 mM sodium phosphate buffer, pH 7.5. Spectra were acquired with data pitch 0.2 nm, averaged over three acquisitions, and smoothed by the Means-Movement algorithm [37].

### Fluorescence Spectroscopy

Protein samples were resuspended in 10 mM sodium phosphate buffer, pH 7.5 and the fluorescence emission spectra were measured on a Cary Eclipse Fluorescence Spectrophotometer (Varian), with excitation at 280 nm and emission range 270–450 nm, employing a 1-cm path-length quartz cuvette. To monitor thermal unfolding, samples were heated from 20 to 95°C at a rate of 1°C/min and fluorescence emission was monitored at 330 nm and at 350 nm. Results are reported as the ratio between the fluorescence emission intensity at 330 nm and at 350 nm to compensate for the fluorescence quenching due to the increasing temperature.

### Fourier Transform Infrared (FTIR) Spectroscopy

FTIR spectra were collected in attenuated total reflection (ATR) using a single reflection diamond ATR device (Golden Gate) and a Varian 670-IR spectrometer equipped with a nitrogen-cooled Mercury Cadmium Telluride detector, under dry-air purging. A sample volume of 3 µl was deposited on the ATR plate in order to obtain a hydrated protein film after solvent evaporation at room temperature [38]. Proteins were measured at a concentration of 2 mg/ml in 10 mM phosphate buffer in experiments performed at 37°C and at a concentration of 1 mg/ml in 50 mM phosphate buffer in experiments performed at 50°C, with the exception of Δα-SpAAP examined at a concentration of 0.4 mg/ml. These buffer conditions were the same used for the activity assays. ATR/FTIR spectra were collected under the following parameters: 2 cm^−1^ spectral resolution, 25 kHz scan speed, 512 scan co-additions, triangular apodization. When necessary, spectra were corrected for the residual water vapor interference. Under these conditions, spectra with excellent signal to noise ratio were obtained, as can be seen by the inspection of their second derivatives in the 1700–1750 cm^−1^ spectral region. The second derivatives [39] of the spectra were obtained by the Savitzky-Golay algorithm (5 points), after an 11-point binomial smoothing of the measured spectra, using the Grams/AI software (Thermo Electron Corporation).

## Results

### *In vivo* Solubility of Recombinant SpAAPs is Affected by Domains Association and by the α1-helix

**Figure 1:**
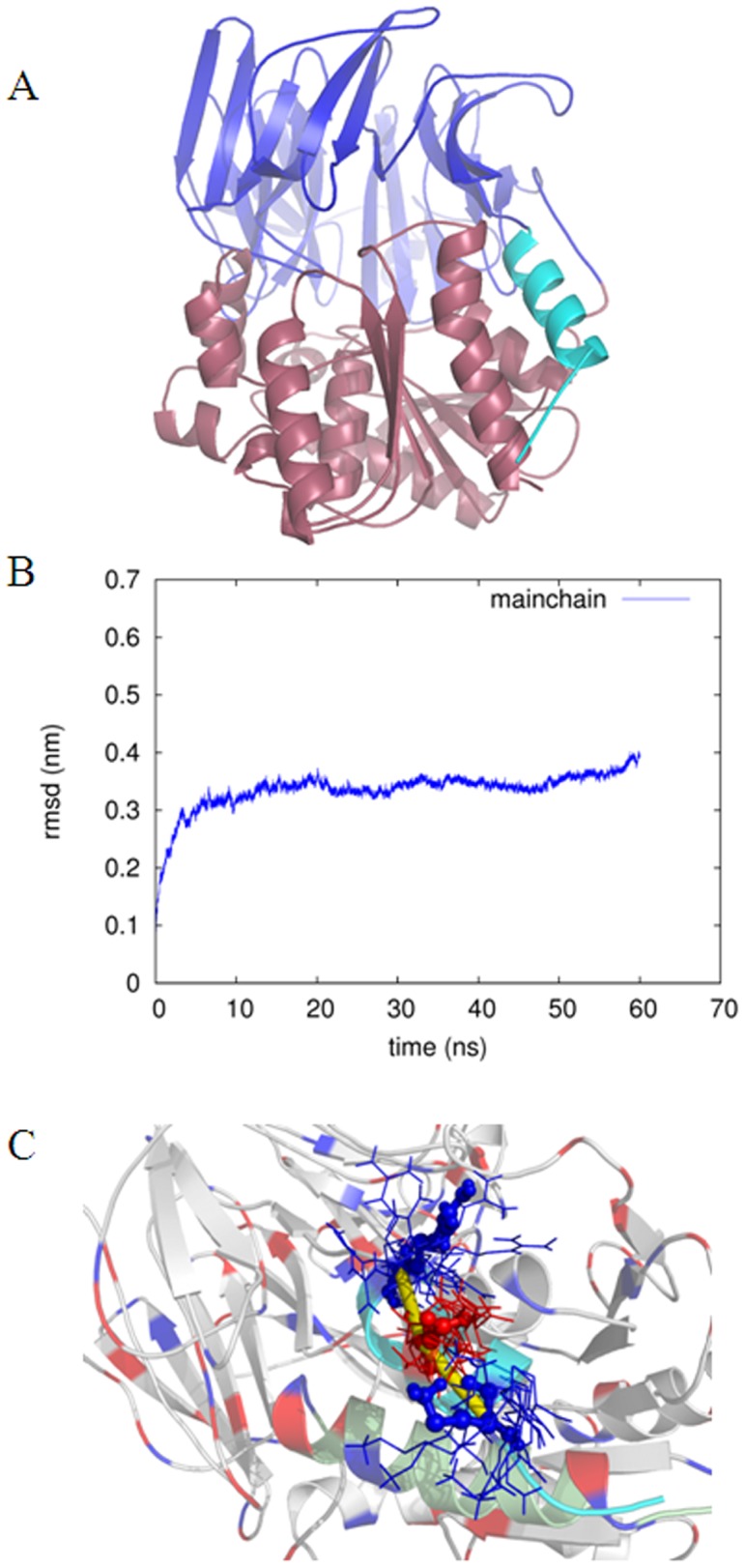
3D structure of SpAAP. (**A**) The 3D structure of SpAAP as derived by homology modeling. The N-terminus (aa 1–332) includes the β-propeller domain (in blue), whereas the C-terminal region (333–696) the catalytic α/β-hydrolase domain (in purple) and the α1-helix (in cyan). (**B**) Time-evolution of the mainchain root mean square deviation with respect to the initial model. (**C**) Structural and dynamics features of the α1-helix. The intra-helical salt bridges involving the three charged residues of the α1-helix are shown as sticks, with thickness proportional to their persistence during the MD simulations. The three mutation sites are shown as sticks in the average structure obtained from the MD, whereas their orientation in other snapshots of the dynamics trajectory is shown as lines. The N-terminal α1-helix is colored in cyan and the C-terminal helix in green.

We have used homology modeling and fold recognition approaches to predict the 3D architecture of SpAAP, whose amino acid sequence was determined in a previous work [23]. This enzyme, as all members of the POP family, is a two-domains protein with an N-terminal portion (α1-helix) which protrudes from the β-propeller domain and folds on the catalytic C-terminal domain [11], [23], [40]. The boundaries between the β-propeller (residues 1–332) and the catalytic domain (333–596) were defined by Pfam [41], a database for structural domain detection (Figure 1A). Inspection of the SpAAP model suggested that the α1-helix involves residues 6–17 (Figure 1A) and MD simulations carried out to refine the model identified the same helix in the region 5–14. To verify convergence of the simulations to stable RMSD values, the main chain root mean square deviation (RMSD) was calculated with respect to the MD initial structures. The first 6 ns of the simulation were required for convergence, and they were therefore discarded for the successive analyses of the MD trajectory (Figure 1B). The distribution of charged amino acids along the α1-helix suggests the possibility of salt-bridge interactions with the C-terminal domain, in particular with the α-helix predicted from residue 577 to 595. However, analysis of salt bridges in the MD ensemble did not evidence any persistent interaction with the C-terminal domain, while it highlighted a high persistence of intra-helical interactions among the three charged residues of α1 (K6, E10 and R14, Figure 1C). Indeed, these residues appear to belong to a three-nodes intra-helical salt-bridge network in which E10 interacts with both K6 and R14, featuring these interactions in more than 70% of the MD frames.

**Figure 2:**
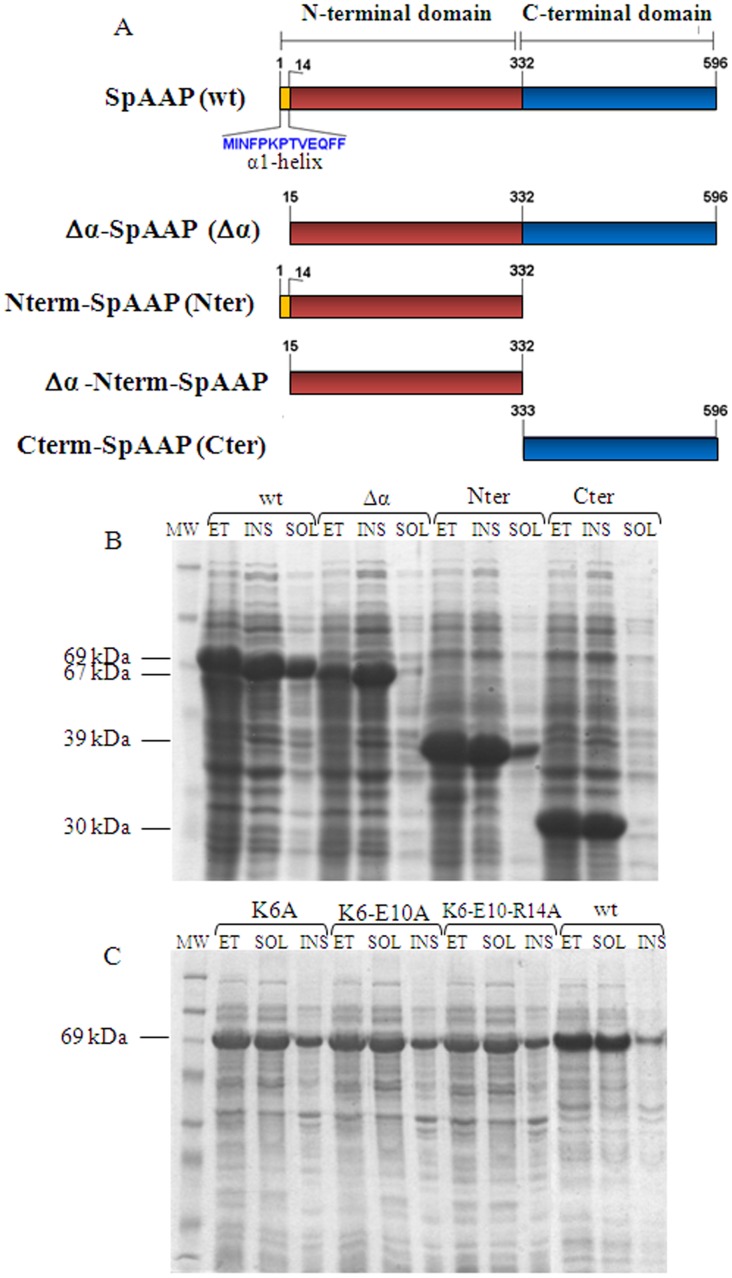
Design of SpAAP variants and analysis of their solubility. (**A**) Domain organization of SpAAP and schematic representation of the deletion mutants analyzed in this study. (**B, C**) SDS-PAGE analysis of solubility of SpAAP variants produced in *E. coli* cells. Frozen cell pellets were handled at room temperature to separate the soluble and insoluble protein fractions. For each sample, total cell extracts (ET), soluble proteins (SOL) and insoluble proteins (INS) were extracted from the same amount of cells (0.12 OD_600_). (**B**) Samples from cells producing the wild-type protein (wt), the protein deleted of the N-terminal helix (Δα), the isolated β-propeller (Nter), the isolated catalytic domain (Cter). (**C**) Samples from cells producing the charge mutants K6A, E10A, R14A. The solubility profile of all single-charge mutants were similar and are not shown. MW: molecular weight marker.

In order to study the relevance of each one of the two domains for the functional properties of the enzyme, we produced them separately through amplification of the two gene segments by back-to-back PCR with the appropriate primers (Table S1) and expression in *E. coli* BL21 cells. A schematic representation of the deletion mutants of this study is reported in figure 2A. Solubility of the protein variants was analyzed on crude cell extracts prepared according to an analytical extraction protocol, at room temperature from frozen cell samples. These experiments revealed that the whole SpAAP protein and the isolated β-propeller domain were largely soluble, while the C-terminal catalytic domain was only found in the fraction containing insoluble proteins (Figure 2B). This observation hints that out of the context of the whole protein, the catalytic domain is structurally unstable and/or unable to fold properly. Accordingly, analysis of the 3D model showed that this domain in isolation exposes hydrophobic and aromatic residues, such as F389, L336 and W498, which in the intact structure are buried at the domains interface and might drive aggregation when not shielded. Moreover destabilization might arise also from the lack of the N-terminal helix that belongs in sequence to the β-propeller and has been reported to stabilize the interactions between the two domains in the homologous ApAAP [10].

On this basis, to assess the relevance of the N-terminal helix we produced a protein deleted of residues 1–14 (Δα-SpAAP) as well as the isolated β-propeller lacking the same stretch of amino acids (Δα-Nterm-SpAAP). Upon expression and analysis of crude extracts as described before, Δα-SpAAP was found in the insoluble fraction only (Figure 2B). Thus, the effect on protein solubility caused by removing the whole N-terminal domain was reproduced by deleting a much smaller protein segment spanning the first 14 residues only. On the contrary, Δα-Nterm-SpAAP was largely recovered in the soluble fraction (data not shown). We should underline that our protocol for analytical extraction, although unsuitable to obtain some of the variants as soluble proteins, proved useful to give a first indication about differences in the stability of protein variants.

In the search of specific amino acids involved in the stabilization of the structure, and considering the scenario depicted by MD simulations, K6, E10 and R14 were substituted with alanine. As a negative control also N3, which is apparently not involved in interactions with other parts of the protein, was mutated. All charge mutants (single position variants, the double mutant SpAAP^K6A−E10A^, and the triple mutant SpAAP^K6A−E10A−R14A^) were obtained in the soluble proteins fraction (Figure 2C).

In order to increase the yield of soluble proteins, we implemented the protocol of extraction and purification by performing all steps at 4°C. Moreover, cell pellets were handled immediately after cells harvesting, avoiding freezing and storage. When applied to C-terminal domain and Δα-SpAAP proteins, this procedure allowed to increase the soluble fraction from undetectable up to 40% of the whole recombinant protein, whereas no changes were observed for other mutants and wild-type SpAAP. All biochemical and biophysical experiments described in the following have been therefore performed with protein samples prepared at 4°C.

### Activity and Kinetic Stability Rely on Domains Association

**Figure 3:**
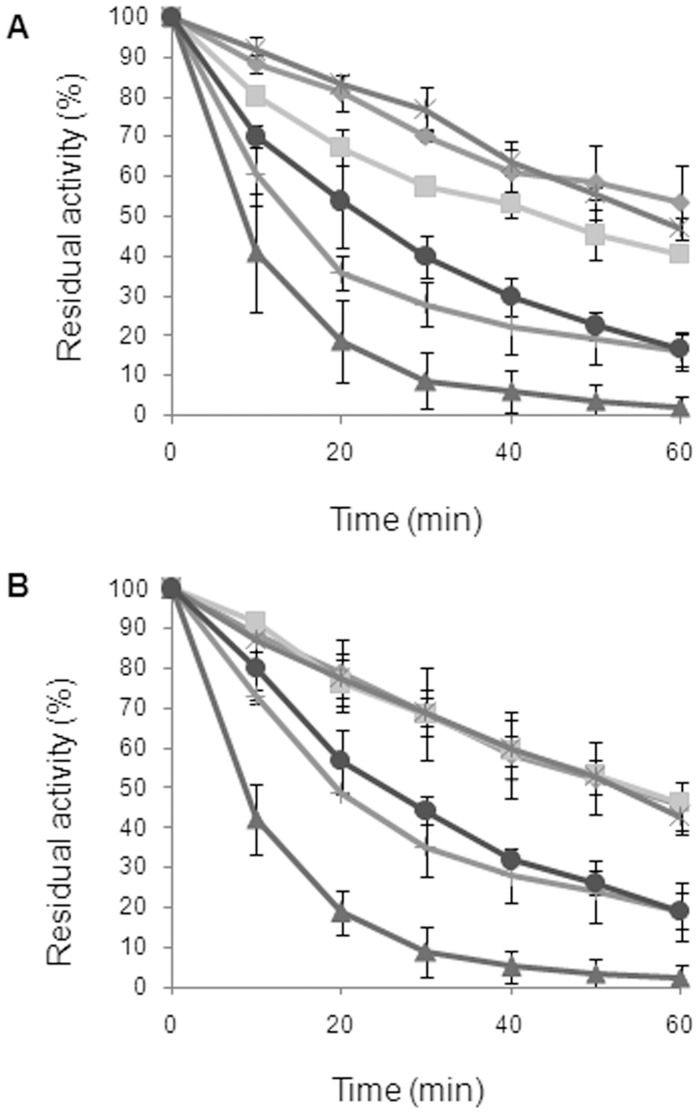
Kinetic stability of SpAAP variants. Freshly purified proteins were incubated at 50°C, before determining residual activities on *p*NP-But (**A**) and Nac-leu-*p*NA (**B**). Activity at t_0_ was taken as 100%. Wild-type SpAAP (diamonds), K6A (squares), E10A (cross plus), R14A (stars), K6A-E10A (triangles), K6A-E10A-R14A (circles). Experiments were carried out in triplicate and error bars presented in the plot.

Proteins were assayed for their enzymatic activity and kinetic stability immediately after purification on both *p*NP-but and Nac-leu-*p*NA (Table 1 and Figure 3). Neither Δα-SpAAP (the whole protein lacking the α1-helix) nor SpAAP-Cterm (the isolated catalytic domain) did exhibit hydrolytic activity in any of the conditions of substrate, temperature and enzyme concentration assayed, notwithstanding they contain a complete α/β-hydrolase domain. Again, these data support the hypothesis that the catalytic moiety of SpAAP cannot exist as an autonomous structural and functional entity, separated from the β-propeller.

**Table 1:** Specific activities (U/mg) of SpAAP variants measured at 25°C on different substrates.

	NAc-leu-pNA	NAc-Phe-pNA
wt	1,372±0,024	1.083±0.053
K6A	1.321±0.044	0.885±0.006
E10A	1.042±0.046	0.870±0.083
R14A	1.160±0.005	0.788±0.029
K6A-E10A	0.888±0.075	0.704±0.040
K6A-E10A-R14A	0.753±0.152	0.515±0.014
	**pNP-But**	**pNP-Cap**
wt	13.71±0.250	2.04±0.026
K6A	10.56±0.425	1.81±0.095
E10A	12.06±0.201	1.52±0.018
R14A	12.70±0.133	1.99±0.005
K6A-E10A	13.72±0.671	1.63±0.333
K6A-E10A-R14A	9.69±0.195	1.18±0.064

Esterase activity was assayed on p-nitrophenyl butyrate (pNP-But) and p-nitrophenyl caprilate (pNP-Cap); peptidase activity was assayed on N-acetyl-L-leucine-p-nitroanilide (Nac-leu-pNA) and N-acetyl-L-phenylalanine-p-nitroanilide (Nac-phe-pNA).

Assays carried out with the whole enzyme and with its variants carrying single or multiple substitutions in the α1-helix showed that the substrate specificity of all mutants was unaltered with respect to the wild-type enzyme (Table 1). This is not obvious since it was suggested that in thermophilic AAP the α1-helix is relevant for the interplay between the domains, the arrangement of the substrate binding site and of the catalytic centre [10], [42], [43]. Our variants, however, differed in their specific activity and kinetic stability. We observed that the specific activity of all single-charge mutants was lower than that of the wild-type enzyme on both substrates. The strongest effect was observed for the triple mutant SpAAP^K6A−E10A−R14A^, whose activity was reduced by ∼45% on the peptide substrates and by 30% on the *p*-nitrophenol esters (Table 1). The kinetics of enzyme inactivation was evaluated at two different temperatures (37°C and 50°C) to catch subtle differences induced by mutagenesis in this fragile protein. At 37°C, a reduction of 15–20% in specific activity was measured for all charge mutants only after over-night incubation (data not shown), whereas incubation at 50°C produced a gradual and similar loss of activity in the wild-type, N3A, K6A and R14A variants, with a decrease of ∼60% within 1 hour of incubation. Exposure to the same temperature dramatically affected E10A, K6A-E10A and K6A-E10A-R14A that, after 20 min lost 65, 85 and 45%, respectively, of their initial activity on *p*NP-But (Figure 3A). We observed that the triple mutant was kinetically more stable than the double mutant.

### The α1-helix Stabilizes the Whole SpAAP but Destabilizes the Isolated β-propeller

**Figure 4:**
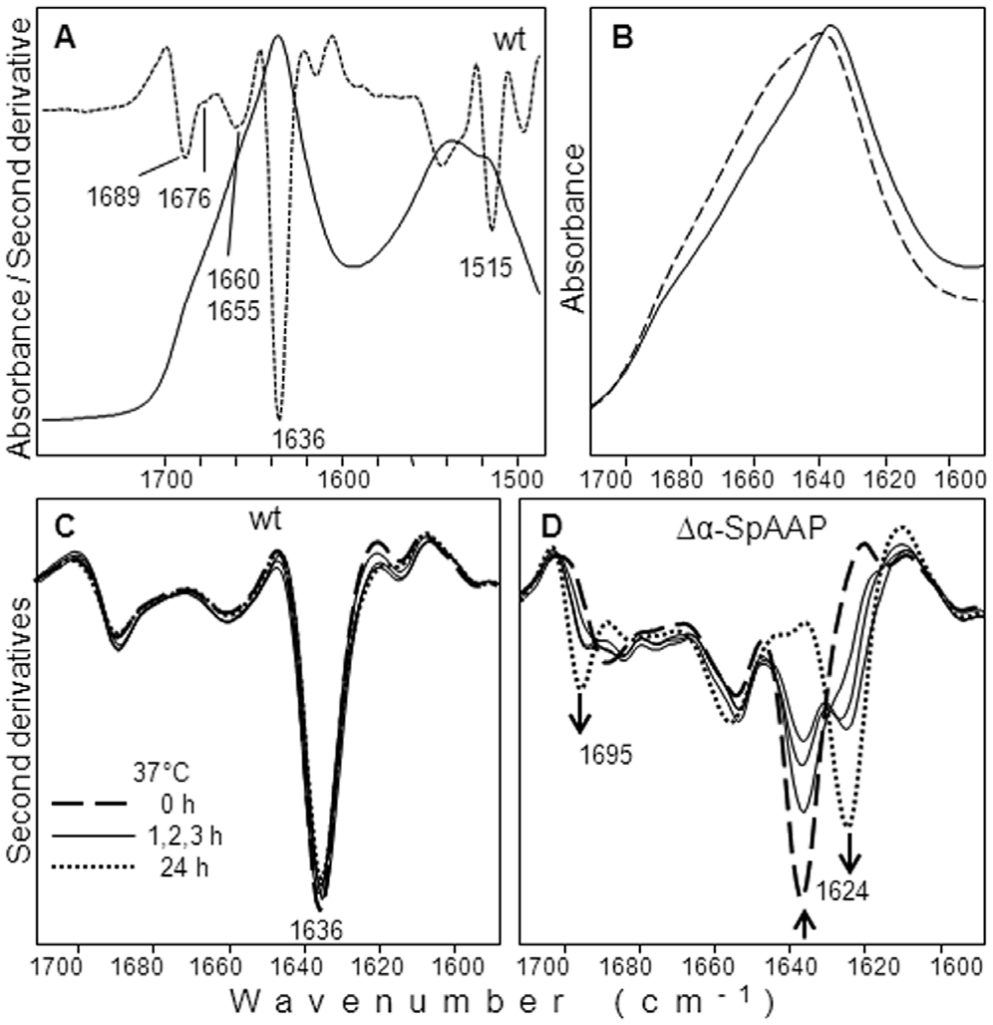
Secondary structure content of SpAAP variants as determined by FTIR spectroscopy. (**A**) The FTIR absorption (continuous line) and second derivative spectrum (dotted line) of freshly purified wild-type SpAAP is reported in the Amide I (1700–1600 cm^−1^) and Amide II (1500–1600 cm^−1^) band region. The spectral region above 1700 cm^−1^ is also given to appreciate the good signal-to-noise ratio in the spectrum. (**B**) FTIR absorption spectra of freshly purified wild-type SpAAP (continuous line) and Δα-SpAAP (broken line). (**C**, **D**) Second derivative FTIR spectra of wild-type SpAAP (**C**) and of Δα-SpAAP (**D**) collected after different times of incubation at 37°C. Arrows point to increased times of incubation.

The conformational stability of wild-type SpAAP and of its variants was investigated by a number of complementary techniques. In FTIR spectroscopy the infrared Amide I band (1700–1600 cm^−1^), due to the C = O stretching vibrations of the peptide bond, is sensitive to the protein secondary structures and to the formation of intermolecular β-sheet structures in protein aggregates [44], [45], [46], [47]. The FTIR spectrum of wild-type SpAAP in 10 mM phosphate buffer is reported in Figure 4A together with its second derivative. This mathematical procedure enables to resolve the different absorption components, due to the protein secondary structures, which are overlapped in the measured absorption spectrum. SpAAP displayed two major components due to the native β-sheet structures of the protein, which appear as negative peaks at ∼1636 cm^−1^ and at ∼1689 cm^−1^ in the second derivative spectrum. Other components were observed at ∼1660 cm^−1^, with a shoulder at ∼1655 cm^−1^, assigned to the α-helices and random coil structures of the protein, and ∼1676 cm^−1^ due to turn structures [45]. Identical results were obtained for the single, double and triple mutants of the N-terminal helix (data not shown), indicating that these mutations do not affect the overall protein secondary structure. On the contrary, Δα-SpAAP displayed reduced intensity of the β-sheet component at ∼1636 cm^−1^ and a higher and broader absorption in the 1660–1650 cm^−1^ spectral region, consistent with a partially unfolded structure (Figure 4B, broken line).

Starting from this information we set up to investigate the effects of deletions and amino acid substitutions on the conformational stability of the proteins by monitoring changes in secondary structure upon incubation at 37°C and 50°C. The C-terminal catalytic domain, though soluble, was produced at low levels and was too unstable for conformational analysis, therefore was not further investigated. As a consequence, the following data refer to the whole protein deprived of the α1-helix, to the β-propeller with or without α-helix and to the single or multiple position variants.

During incubation at 37°C, the IR spectrum kept almost unchanged up to 24 hours for the wild-type protein (Figure 4C) and for most mutants (data not shown), with the only exception of Δα-SpAAP that displayed a rapid decrease of the native β-sheet component at ∼1636 cm^−1^, and a simultaneous increase of two new bands at ∼1624 cm^−1^ and at ∼1695 cm^−1^, assigned to intermolecular β-sheets in the formed protein aggregates (Figure 4D, dotted line). These results indicate that deletion of the α1-helix strongly affects the stability of the psychrophilic AAP, which unfolds and aggregates within a few hours also at mild temperature. Accordingly, in CD analysis Δα-SpAAP showed the strongest propensity to aggregate at 37°C, as witnessed by the loss of the ellipticity signal at 195 nm and the raise of that at 220–222 nm (data not shown). This protein unfolds within a few minutes upon incubation at 50°C.

**Figure 5:**
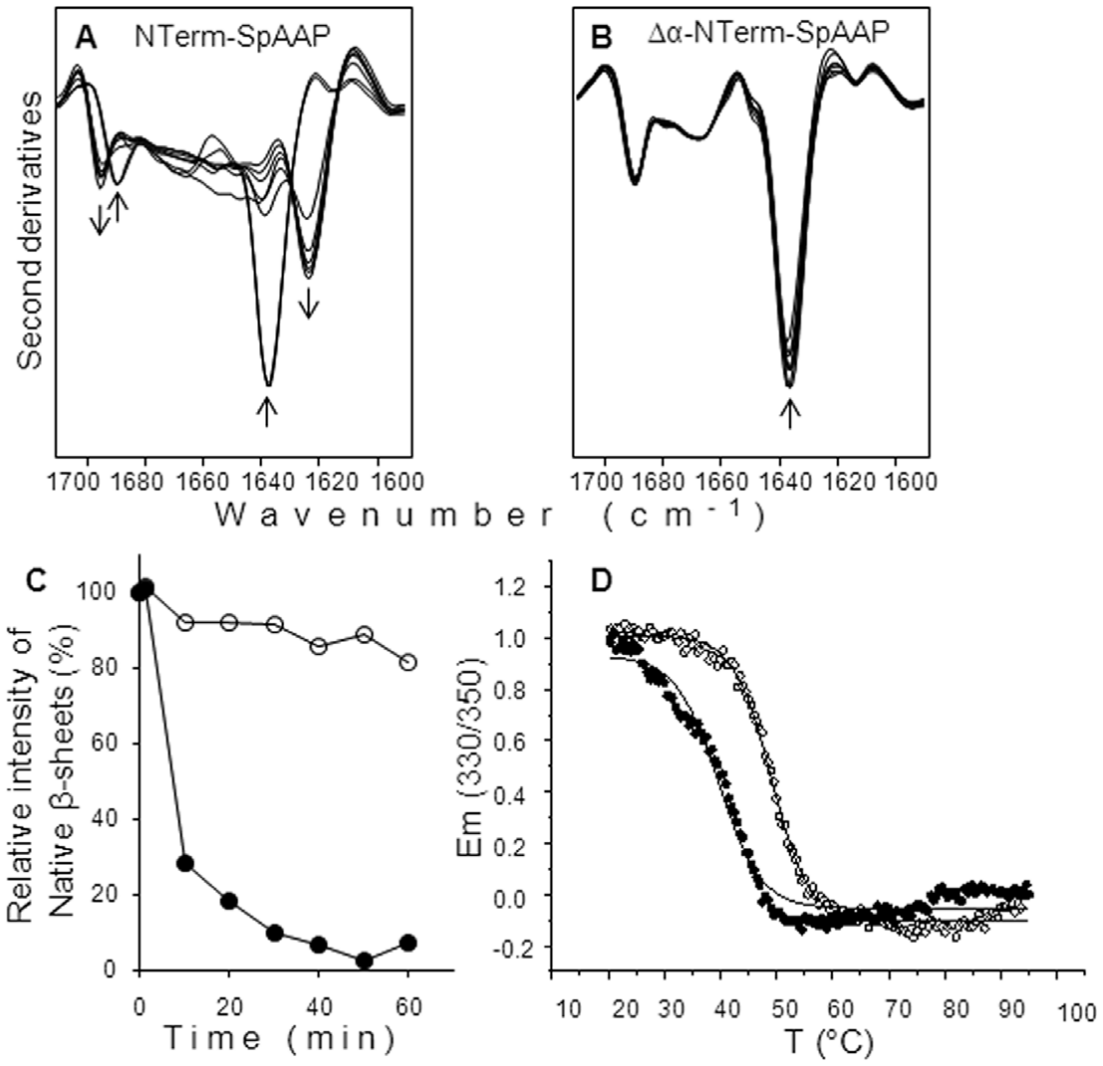
Thermal stability of Nterm domain investigated by FTIR and intrinsic fluorescence analyses. FTIR second derivative spectra of Nterm-SpAAP (**A**) and Δα-Nterm-SpAAP (**B**) collected at different times of incubation at 50°C. Arrows point to increased times of incubation. (**C**) Time dependence of the intensity variation of the native β-sheet component at ∼ 1636 cm^−1^, taken from the second derivative peak-intensity of Nterm-SpAAP (filled circles) and Δα-Nterm-SpAAP (empty circles). (**D**) Thermal denaturation of Nterm-SpAAP (filled circles) and Δα-Nterm-SpAAP (empty circles) was monitored from 20°C to 95°C by intrinsic fluorescence (excitation at 280 nm) and fitted to a two-state model.

Exposure for 1 hour to this challenging temperature (50°C) was useful to highlight differences in the conformational stability of the other mutants (deletion and charge variants of the α1-helix) by FTIR spectroscopy and intrinsic fluorescence. At the beginning of the experiment (measures at time 0 are carried out at room temperature) the IR spectra of Nterm-SpAAP and Δα-Nterm-SpAAP proteins were dominated by the ∼1635 cm^−1^ and ∼ 1689 cm^−1^ peaks, both due to their content of native β-sheets, and are superimposable to the spectra of the wild-type protein. The additional band detected at around 1666 cm^−1^ was assigned to turn structures (Figure 5A, B). These data are well in agreement with the information about the secondary structure recorded by CD in the far-UV at room temperature (data not shown). In the course of incubation, we monitored a fast unfolding of Nterm-SpAAP that lost 80% of the native β-sheet component within 20 minutes. Protein unfolding led to the formation of aggregates, as indicated by the increase of intermolecular β-sheet components detected around 1623 cm^−1^ and 1695 cm^−1^ (Figure 5A). On the contrary, deletion of the α1-helix from the isolated β-propeller resulted in a strong increase of the protein stability. Indeed, Δα-Nterm-SpAAP retained most of its native β-sheet content after 1 hour incubation at 50°C (Figure 5B). We confirmed this unexpected result by studying the variation in the intensity of the protein intrinsic fluorescence upon heating from 20°C to 90°C (Figure 5D). The melting temperatures observed for Nterm-SpAAP (∼39.63°C) and for Δα-Nterm-SpAAP (∼49.06°C) clearly point out their different stability, what suggests that the α1-helix destabilized the β-propeller when it is not associated with the catalytic domain.

**Figure 6:**
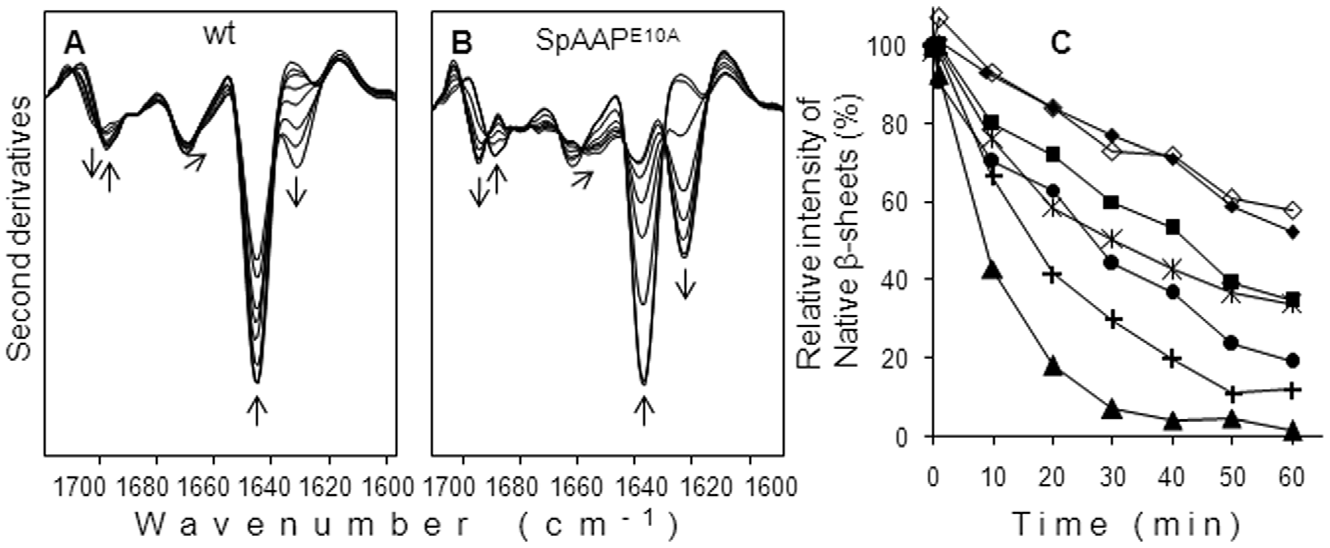
FTIR analyses of SpAAP variants incubated at 50 °**C.** FTIR second derivative spectra of wild-type SpAAP (**A**) and SpAAP^E10^ (**B**), collected at different time of incubation at 50°C. Arrows point to increased times of incubation. (**C**) Time dependence of the intensity variation of the native β-sheet component at ∼1636 cm^−1^, taken from the second derivative peak intensity, for the SpAAP variants: wild-type SpAAP (filled diamonds), SpAAP^K6^ (squares), SpAAP^E10^ (cross plus), SpAAP^R14^ (stars), SpAAP^K6−E10^ (triangles), SpAAP^K6−E10−R14^ (circles), SpAAP^N3^ (empty diamonds).

FTIR second derivative spectra collected at different time of incubation at 50°C for wild-type SpAAP (Figure 6A) were compared with those obtained for charge mutants. Among these latter, only the spectra obtained for SpAAP^E10^ were shown (Figure 6B). All charge mutants displayed a decrease of the IR native β-sheet components at 1636–1635 cm^−1^ and at ∼1689 cm^−1^ and a parallel increase of the aggregates (bands around 1624 cm^−1^ and 1695 cm^−1^) upon treatment at 50°C but the extent of this transition was different depending on the amino acid substitution. During the first hour of incubation at 50°C, wild-type SpAAP and SpAAP^N3A^ progressively lost ∼40% of their initial native β-sheet content (estimated as peak-intensity of the ∼1636 cm^−1^ band) while SpAAP^K6A^ and SpAAP^R14A^ were slightly less stable (Figure 6C). Noteworthy, all proteins carrying the E10 substitution (SpAAP^K6AE10A^, SpAAP^E10A^, and SpAAP^K6A−E10A−R14A^) displayed the fastest unfolding and aggregation kinetics (Figure 6C). Already after 20 minutes at 50°C, the native β-sheet component at ∼1636 cm^−1^ of SpAAP^E10A^ decreased in intensity by ∼60%, disappearing completely with time. In agreement with the kinetics of inactivation, conformational analysis confirms that the double mutant K6A-E10A is the less stable among all variants investigated, and can partly rescue its robustness if R14 is removed from this background.

## Discussion

### Isolated β-propeller and C-terminal Domains of SpAAP are Structurally Unstable and Catalytically Inactive

Previous studies on the role of the two domains in proteins of the POP family suggested that the β-propeller is structurally stable and might regulate the access of the substrates to the active site and therefore the enzyme specificity [17]. It was also hypothesized that the isolated C-terminal domain should retain catalytic activity and be able to accept larger peptides [15]. In our study, the catalytic moiety isolated for the first time from the cold-adapted AAP appears to be structurally unstable and inactive. Moreover, also the β-propeller, which is supposed to be very robust because of its toroidal fold, is less stable than the whole protein. This observation surmises that the interaction between the two protein moieties fulfils unavoidable requirements for the preservation of the reciprocal structural integrity and witnesses their co-evolution.

A major factor contributing to weaken the isolated C-terminal domain might be the exposition to the solvent of hydrophobic and aromatic residues that are usually located at the interdomain surface. According to this view, one could expect that, beside the loss of the whole N-terminal domain, also minor changes that unmask hydrophobic surfaces would cause structural destabilization. In this perspective, we focused our attention on the α1-helix, since the N-terminus – though varying in length and organization among different protein homologues – provides the connection between the two domains in this family of proteins. In the hyperthermophilic ApAAP, deletion of this structure reduced the stability of the protein (decrease of the Tm from ∼115°C to 91°C), but did not hinder the enzyme from maintaining its 3D architecture and activity [10]. In SpAAP however, deletion of the α1-helix produces the same conformational effects observed when the whole N-terminal domain is removed. On the contrary, deleting the same helix from the isolated β-propeller domain produced a strong gain of stability (increase of Tm from 39.62°C to 49.06°C) that we interpret as related to the high mobility of this structure in the absence of the C-terminal domain to which it is anchored through non covalent interactions. In the absence of the catalytic domain, movements of the α1-helix are not restricted (it does not physically interacts with the β-propeller) and unfolding may initiate.

While our results are consistent with those described by Polgár and colleagues in their work on the prolyl oligopeptidases from porcine brain [17] showing that an isolated non-velcroed β-propeller can be quite stable, even when its termini are not embedded into the α/β-hydrolase domain, the stabilization of the propeller through the removal of an α-helix at its N-terminus is unique to the cold-adapted AAP. On this basis we would argue that stabilization of the enzyme structure is achieved by two overlapping mechanisms: bridging the two domains to shield hydrophobic residues and prevent aggregation, and anchoring a loose end to improve conformational stability. Structural information available on AAPs is still poor and is not sufficient to support a general interpretation also because we can compare with some detail only two enzymes very different in their properties, one being hyperthermophilic and thermostable and the other one psychrophilic and thermosensitive. A larger set of examples will allow to conclude whether the paramount relevance of interdomain interactions observed in the cold-adapted protein does depend on its higher flexibility.

### Charged Residues of the α1-helix Contribute to the Overall Protein Stability

Removal of charged residues from the α1-helix destabilizes the enzyme, increasing its sensitivity to thermal inactivation and propensity to aggregate. This result is rather unexpected since salt bridges involving this helix have been shown to be poorly relevant for the stability of the homologous hyperthermophilic ApAAP [10]. On the other hand, the significant loss of stability induced by the double substitution E10A–K6A strongly supports the hypothesis that perturbations of the electrostatic potential between charged residues of the α1-helix interfere with short-range interdomain interactions between the α1-helix and the polar surfaces of the C-terminal domain. It remains to be investigated why the triple mutant is more stable and active than the double-substituted variant, a counterintuitive result in a scenario where each single charge is hypothesized to contribute to the intradomain and interdomain stabilization of the whole structure. Our model fails to point out persistent interactions among charged residues of α1-helix and the C-terminal domain. We are aware of the limitation in the use of the model imposed by the low sequence identity of SpAAP with the template ApAAP. A different experimental approach, based on the mutations predicted by a wider phylogenetic analysis and ancestral inference [48] could help to find interacting residues as possible targets for compensatory, stabilizing mutations.

The good correspondence of the inactivation curves and the aggregation plots derived by FTIR spectra suggests that the loss of interdomain interaction does affect the whole structure of the enzyme, including its catalytic core. Only in the case of the R14A protein, structural collapse seems to precede inactivation. This effect of uncoupling between activity and stability could be explained assuming that the catalytic center remains relatively protected from the structural effects produced by the mutation R14A. Indeed, we cannot exclude that charged residues in the α1-helix could mediate interactions between the N-terminal helix and the catalytic site. Such a long-range effect has been recently investigated in a computational work on ApAAP suggesting that hydrophobic residues of the α1-helix might concur to the maintenance of the functional architecture of the catalytic triad [42].

We do not underestimate the possible contribution of other interactions between the two protein domains. For instance, an interdomain salt bridge between Glu88 and Arg526 was demonstrated to play a major role for both the stability and the substrate specificity of ApAAP [49], while the existence of hydrophobic interactions is suggested by the analysis of the interface between the catalytic domain and the α1-helix in our model of SpAAP (present work, data not shown). Indeed, I581 and Y584, which interact with the hydrophobic residues F12 and F13 of the α1-helix, become exposed to the solvent when the N-terminal α1-helix is removed. Overall (and with some caution due to the lack of an experimental 3D structure), our work hints the involvement of the α1-helix in a complex network of hydrophobic and polar interactions that could be investigated in the next, even by assessing the stability of the α1-helix itself.

It is worth to underline that in AAPs intradomain communication also mediates the reciprocal movements allowing substrates to enter the binding site and to reach the catalytic centre. As a consequence, it could be expected that a loose connection between the N- and C-terminal domains would allow the access of larger molecules to the substrate binding site. However, the observation that the profile of substrate specificity remains unchanged for all the mutants obtained in this study supports rather the view that substrate specificity is finely determined by the positions of amino acid residues at the substrate binding site and not simply by the strength of the contact between the two domains.

In the overall picture of this family of intracellular proteases it is reasonable to consider that the structural mechanism for substrate selection should be tightly controlled and that the loss of stability consequent to the enlargement of the substrate accessibility is part of a regulation strategy directly implying the structural crash of the enzyme. Structural elements that guarantee a tight and regulated association between the two domains might have been subjected to strong evolutionary pressure. One can assume that for a cell would be preferably to manage with the loss of enzyme molecules, rather than with an intracellular proteolytic activity out of control.

## Supporting Information

Table S1 **Sequence of oligonucleotides used for mutagenic PCR.**
(DOC)
