# Supplementary material for: Reciprocal Influence of Protein Domains in the Cold-Adapted Acyl Aminoacyl Peptidase from Sporosarcina psychrophila
Source: PLoS One. 2013 Feb 15;8(2):e56254. doi: 10.1371/journal.pone.0056254 (PMC3574126; doi:10.1371/journal.pone.0056254)
Supplement: Table S1 — Sequence of oligonucleotides used for mutagenic PCR. (DOC) [file pone.0056254.s001.doc]

**TABLE S1**

Sequence of oligonucleotides used for mutagenic PCR

| **Oligo Name** | **Sequence (5’-3’)** | **Template** | **Product** | |
| --- | --- | --- | --- | --- |
| C-term.for | ATGGTCTTGGGATTAAGTCCAGAGG | pET22[SpAAP] | pET22[SpAAP_Cterm] |  |
| C-term.rev | ATGTATATCTCCTTCTTAAAGTTAAACAAAATTATTTCTAG |  |
| Δα.for | ATGACGTATACGATTACAAACTTTGCAGTAAG | pET22[SpAAP] | pET22[SpAAP_Δα] |  |
| Δα.rev | ATGTATATCTCCTTCTTAAAGTTAAACAAAATTATTTCTAG |  |
| N-term.for | GAGCACCACCACCACCAC | pET22[SpAAP] | pET22[SpAAP_Nterm] |  |
| N-term.rev | GAGACGATTGTTCGTCAATTGCTTCC |  |
| ΔαN-term.for | ATGACGTATACGATTACAAACTTTGCAGTAAG | pET22[SpAAP_Nterm] | pET22[SpAAP_ ΔαN-term] |  |
| ΔαN-term.rev | ATGTATATCTCCTTCTTAAAGTTAAACAAAATTATTTCTAG |  |
| N3A.for | AATTTTTTCGAACGTATACGATTACAAAC | pET22[SpAAP] | pET22[SpAAP^N3A^] |  |
| N3A.rev | GCTCGACTGTTGGTTTCGGAAACGCAATC |  |
| K6A-E10A.for | AATTTTTTCGAACGTATACGATTACAAAC | pET22[SpAAP] | pET22[SpAAP^K6A_E10A^] |  |
| K6A-E10A.rev | GCGCGACTGTTGGCGCCGGAAAATTAATC |  |
| R14A.for | AATTTTTCGCGACCTATACGATTACAAAC | pET22[SpAAP^K6A_E10A^] | pET22[SpAAP^R14A^] |  |
| R14A.rev | GCTCGACTGTTGGTTTCGGAAAATTAATC |  |
| E10A.for | TTTCGAACCTATACGATTACAAACTTTGC | pET22[SpAAP] | pET22[SpAAP^E10A^] |  |
| E10A.rev | AAATTGCGCGACTGTTGGTTTCGG |  |
| K6A.for | TTTCGAACCTATACGATTACAAACTTTGC | pET22[SpAAP] | pET22[SpAAP^K6A^] |  |
| K6A.rev | AAATTGCTCGACTGTTGGCGCC |  |
| K6A-E10A-R14A.for | AATTTTTCGCGACCTATACGATTACAAAC | pET22[SpAAP^K6A_E10A^] | pET22[SpAAP^K6A_E10A_R14A^] |  |
| K6A-E10A-R14A.rev | GCGCGACTGTTGGCGCCG |  |
